# Clinical outcome after lumbar spinal fusion surgery in degenerative spondylolisthesis: a 3-year follow-up

**DOI:** 10.1007/s00402-020-03697-9

**Published:** 2020-12-29

**Authors:** Maximilian Lenz, S. Oikonomidis, R. Hartwig, R. Gramse, C. Meyer, M. J. Scheyerer, C. Hofstetter, P. Eysel, J. Bredow

**Affiliations:** 1 Department of Orthopaedic and Trauma Surgery, University Hospital of Cologne, Kerpener Str. 62, 50937 Cologne, Germany; 2 Department of Neurological Surgery, UW Medicine Seattle, Seattle, WA USA

**Keywords:** Spondylolisthesis, Fusion surgery, Reduction, Sagittal balance, Clinical outcome

## Abstract

**Introduction:**

Lumbar spinal fusion surgery is a widely accepted surgical treatment in degenerative causes of lumbar spondylolisthesis. The benefit of reduction of anterior displacement and restoration of sagittal parameters is still controversially debated. Purpose of the underlying publication was to analyze the influence of radiographic sagittal parameters of the spine in aspects of changes in postoperative clinical outcome.

**Materials and methods:**

By prospective analysis, we included patients with low-grade degenerative lumbar spondylolisthesis (Meyerding grades I and II) with mono- or bisegmental fusion surgery with a minimum follow-up data of 3 years. For clinical outcome measures, COMI, ODI and EQ-5D were used. Spinopelvic parameters (sacral inclination, pelvic tilt, sacral slope and pelvic incidence, lumbar lordosis and lumbar index as well as anterior displacement and sagittal rotation) were measured on plain radiographs.

**Results:**

We could observe a significant benefit in clinical outcome after lumbar fusion surgery in low-grade spondylolisthesis in our mid-term follow-up data including 32 patients. By surgical reduction, we could see significant restoration of anterior displacement and sagittal rotation. Interestingly, a significant correlation between restoration of both sagittal rotation and sacral inclination and clinical outcome score was observed in the 3-year follow-up.

**Conclusion:**

In low-grade spondylolisthesis, spinal fusion surgery is a well-established surgical procedure; however, the impact of sagittal parameters and reduction of anterior displacement remains controversial. Within our findings, restoration of sagittal parameters showed significant correlation to improvement in clinical outcome in our mid-term follow-up data.

## Introduction

Degenerative lumbar spondylolisthesis is a commonly seen cause of severe low back pain with incidence levels described of around 6% [1]. Mostly patients older than 50 years are affected with women showing faster rate of developing spondylolisthesis [2]. Low-grade degenerative spondylolisthesis (Meyerding grades I and II) can be treated primarily by conservative therapy [3]. However, with persisting severe low back pain, operative therapy must be evaluated. Lumbar spinal fusion surgery is a widely accepted surgical treatment and is performed either by posterior or transforaminal lumbar interbody fusion (PLIF/TILF) [4, 5].

Numerous factors can influence the clinical outcome in spinal surgery [6, 7]. The sagittal balance of spine and pelvis is supposed to be one of the factors that influence the clinical outcome in spinal fusion surgery [8, 9]. In radiographic analysis, patients suffering from degenerative spondylolisthesis show higher sacral slope and pelvic tilt values resulting in sagittal imbalance and consecutively pelvic compensation [10–13].

However, clinical outcome following lumbar spinal fusion in cases of degenerative low-grade spondylolisthesis is varying and the impact of the sagittal parameters on postoperative outcome remains controversial due to a paucity of studies. Especially, the benefit of reduction of anterior displacement of the spine and restoration of sagittal parameters is still debated. Furthermore, other factors such as grade of spondylolisthesis (anterior displacement) or surgical technique (fusion and decompression or simple decompression without fusion) are also controversially discussed [14, 15]. So far, several studies have analyzed the sagittal rotation and anterior displacement of the affected segment and the impact on clinical outcome in spinal surgery. Within these findings, no significant benefit was postulated in regards of clinical outcome in reduction and restoration of sagittal parameters [16]. In contrary, other findings imply an improvement in clinical outcome after reduction surgery [17].

Thus, the aim of the presented study was to investigate the mid-term clinical outcome after lumbar spinal surgery in degenerative spondylolisthesis and the impact of restoration of the sagittal balance and anterior displacement. Therefore, radiological spinopelvic sagittal parameters, anterior displacement of the affected segment and reduction of sagittal rotation in lumbar spinal fusion surgery were analyzed in our patients’ group within a 36 months’ follow-up period.

## Materials and methods

### Study design

We performed a prospective study, including patients with low-grade degenerative lumbar spondylolisthesis who underwent lumbar spinal surgery. Lumbar fusion surgery was performed in open procedure by three different senior orthopedic surgeons for symptomatic low-grade spondylolisthesis using pedicle screw fixation, partial laminectomy, disc removal and cage insertion. Either PLIF or TLIF surgery of mono- or bi-segmental levels was included according to patient’s pathology within 2013 and 2015 for symptomatic low-grade spondylolisthesis. PLIF and TLIF were performed open using pedicle screw fixation, partial laminectomy, disc removal and cage insertion. TLIF was performed in cases of mild spondylolisthesis (Meyerding I) and only unilateral stenosis of neuroforamina. A minimum of 3-year follow-up and examination data had to be available. Criteria of exclusion were development of multisegmental fusion surgery, osteoporosis, suffering of tumorous or infectious diseases, spinal fractures and neurological deficits, preoperatively. Written consent was obtained of all participants and the ethics committee of our institution approved our study (No. 09-182).

### Data collection

The data were extracted of the Spine Tango Registry such as gender, age, body mass index as well as length of stay, surgical time and peri- or postoperative complications. Patients were examined at 12, 24 and 36 months of follow-up.

### Clinical outcome measures

Questionnaires such as the Core Outcome Measure Index (COMI), the European Quality of Life 5 Dimensions (EQ-5D) and the Oswestry Disability Index (ODI) were used to obtain objective clinical outcome data. The presented questionnaires are recommended by the German Spine Society (DWG) and European Spine Society (Eurospine) for outcome measurements.

### Radiological measurements

**Fig. 1:**
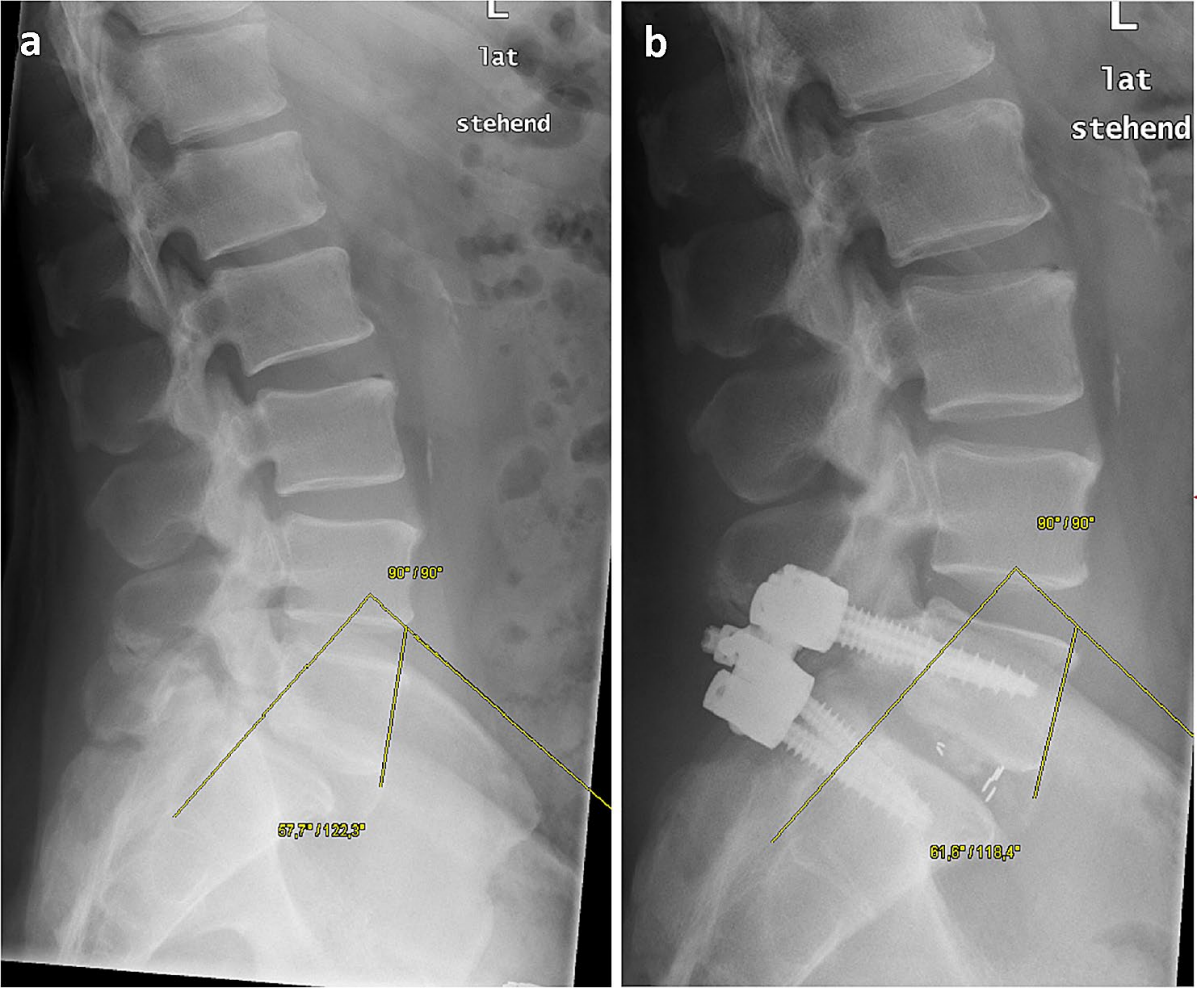
**a**, **b** Measurement of sagittal rotation preoperatively (**a**) and after PLIF L5/S1 (**b**). The angle **a**: 57.7; **b**: 61.6°) is determined by extending a line along the anterior border of the body of the fifth lumbar vertebra until intersecting a line along the posterior border of the body of the first sacral spinal body

Standardized conventional radiography in two planes in standing position was performed pre- and postoperatively and at time of follow-up examination. Radiological measurements were performed by one senior orthopedic resident and single measurement for each parameter was performed. Spinopelvic parameters (sacral inclination, pelvic tilt, sacral slope and pelvic incidence), lumbar lordosis and lumbar index were measured. Anterior displacement (slippage) and sagittal rotation were defined and measured as shown in Fig. 1 [18]. Boxall’s et al. [19] technique was applied to evaluate the degree of anterior displacement, determining the ratio of percentage of anterior displacement and regarding the length of the vertebral body. To classify the grade of spondylolisthesis, the Meyerding Classification was used preoperatively.

### Statistical analysis

For statistical analysis, SPSS Software (IBM SPSS Statistics Version 25, 76 Chicago, IL, USA) was used. Student’s t test for paired samples was used to compare the mean parameters of the clinical and radiological. Using the Kolmogorov–Smirnov test, normal distribution for pre- postoperative data was obtained. Significance level of p values was set as of *p* < 0.05. To evaluate the correlation of the radiological parameters with clinical findings, a correlation analysis was performed using the Spearman-Rho bi-serial test. For the correlation analysis, the change of each radiological parameter preoperative to postoperative follow-up was correlated with the difference of the clinical outcome.

## Results

### Clinical outcome

A total of 32 patients (19 females and 13 males) matched the criteria of the 3-year follow-up and were included in this study. Initially included were 62 patients and 30 were lost from 2- to 3-year follow-up. TLIF was performed within three patients and PLIF in 29 patients. The mean age of all patients accounted for was 59.2 ± 15.3 years with 23 having had monosegmental fusion and nine patients with bisegmental fusion surgery. Of the monosegmental fusions, 11 subjects had fusion of the L4/5 segment, another 11 subjects had fusion of L5/S1 and one patient of L3/4, respectively. When performing bisegmental fusion, five subjects had fusion of L4/5, L5/S1, two subjects had fusion of segments L3–5 and one had fusion of segments L2–4. Main indication for bisegmental fusion surgery was highly degenerative spondylolisthesis with instability and associated osteochondrosis.

Mean BMI level was 28.04 ± 6.7 (kg/m^2^) (range 18.4–45). Mean time of operation was 186.74 ± 58.9 min. (range 110–379) and mean blood loss levels were 834 ± 542.6 ml (range 200–2000). Moreover, two cases of intraoperative dural leakage were observed. Mean length of hospital stay was 14.4 ± 4.2 days (range 7–24). Postoperative complications were observed in seven cases, with only one patient in need for revision surgery due to postoperative implant dislocation.

Preoperatively, mean COMI score was 8.1 ± 1.2 (range 5.54–10.00) mean ODI was 47.6 ± 18.3; (range 16.0–84.0) and mean EQ-5D was 0.29 ± 0.34 (range − 0.25 to 0.8). When performing the 3 years’ follow-up examination, postoperative mean COMI score was 4.3 ± 2.3 (range 0.0–9.6), the mean ODI was 26.0 ± 18.5 (range 2.0–60.0) and mean EQ-5D score was 0.70 ± 0.27 (range − 0.24 to 1.00) respectively (Table 1). Thus, in regards of the clinical outcome after lumbar fusion surgery, a significant change in pain scores was obtained at 3 years of follow-up (*p* = 0.000).

**Table 1:** Comparison of clinical measurements with preoperative data and results in the 36 months follow-up

	Preoperatively		3-year follow-up	
COMI	8.2 ± 1.3 (range: 5.5–10.0)	*p* = 0.00	4.3 ± 2.3 (range: 0.0–9.6)	*p* = 0.00
ODI	51.2 ± 19.2 (range: 11.0–98.0)	*p* = 0.00	26.00 ± 18.5 (range: 0.0–60.0)	*p* = 0.00
EQ-5D	0.27 ± 0.35 (range: − 0.6 to 0.8)	*p* = 0.00	0.7 ± 0.27 (range: − 0.2 to 1.0)	*p* = 0.00

### Radiological outcome

According to the radiological data, 26 patients suffered preoperatively from degenerative low-grade spondylolisthesis with mean anterior displacement of 18.88 ± 12.79 (Table 2). Mean value at the 3 years’ follow-up for pelvic incidence was 62.02° ± 13.62°, mean pelvic tilt was 23.31° ± 8.34° and mean sacral slope was 39.43° ± 15.06°. Significant change was found in sacral inclination, anterior displacement and sagittal rotation (Table 2).

**Table 2:** Radiological data shown with mean values, standard deviation and minimum and maximum values with both preoperatively and at time of 36 months of follow-up

Preoperative	Mean	Std.Dev	Min	Max	36-month follow-up	Mean	Std.Dev	Min	Max	*P* values
Gliding angle	11.33	± 5.94	0.6	24.8	Gliding angle	10.72	± 6.17	4	23	*p* = 0.756
Sacral inclination	46.72	± 10.72	14.1	66.9	Sacral inclination	42.89	± 6.61	28.8	50.1	*p* = 0.032
Anterior displacement	18.88	± 12.79	1.5	49.5	Anterior displacement	11.94	± 8.17	1.9	34.7	*p* = 0.000
Sagittal rotation	67.14	± 8.13	47.1	88.6	Sagittal rotation	71.54	± 6.42	64	89.2	*p* = 0.000
Lumbar index	82.96	± 8.93	61.5	96.5	Lumbar index	84.00	± 7.47	71.9	97.1	*p* = 0.069
Lumbar lordosis	42.99	± 14.67	14.5	81.4	Lumbar lordosis	41.95	± 17.11	6.7	68.1	*p* = 0.857
Sacral slope	38.19	± 9.02	18.2	53.1	Sacral slope	39.43	± 15.06	8.6	84.5	*p* = 0.204
Pelvic tilt	22.21	± 7.96	9.7	42.4	Pelvic tilt	23.31	± 8.34	10.9	35.9	*p* = 0.154
Pelvic incidence	61.21	± 12.35	33.5	91.7	Pelvic incidence	62.02	± 13.62	40	85.9	*p* = 0.879

Furthermore, mean lumbar lordosis was assessed with 42.99° ± 14.67° preoperatively, concluding a lumbar index (lumbar lordosis/pelvic incidence) of 82.96 ± 8.93. At follow-up, mean value for lumbar lordosis was 41.95° ± 17.11 with a lumbar index of 84.0 ± 7.47 (see Table 2) of 36 months of follow-up. By surgical reduction and restoration of the lumbar spine, the preoperative anterior displacement was significantly reduced from 18.88 ± 12.79 to 11.94 ± 8.17 (Table 2). This significant reduction was observed directly postoperatively and was retained during period of follow-up (*p* = 0.000).

### Correlation of radiological parameters and clinical outcome

We found correlation between postoperative clinical outcome and radiological measurements of patients with degenerative spondylolisthesis. In detail, a strong correlation of COMI score with both, sacral inclination (r*0.586) and sacral slope (r*0.532) was observed and is demonstrated in Table 3. Furthermore, a strong correlation of ODI and sagittal rotation was seen in patients after 36 months of follow-up (r* 0.517; *p* < 0.05). Regarding all the other radiological parameters analyzed in this study, no further significant correlation with clinical outcome in COMI, ODI or EQ-5D could be detected (Table 3).

**Table 3:** Correlation of clinical and radiological parameters at 36 months of follow-up after surgery (*n* = 32) showing a strong correlation

	Statistics	COMI	ODI	EQ-5D
Gliding angle	Spearman’s Rho	0.029	− 0.213	0.059
	Bivariant	0.919	0.447	0.835
Sacral inclination	Spearman’s Rho	*− 0.586*	− 0.213	0.163
	Bivariant	**0.022**	0.447	0.562
Anterior displacement	Spearman’s Rho	− 0.171	− 0.038	0.102
	Bivariant	0.541	0.894	0.718
Sagittal rotation	Spearman’s Rho	0.35	*0.517*	− 0.347
	Bivariant	0.201	**0.049**	0.205
Lumbar index	Spearman’s Rho	− 0.111	0.161	− 0.118
	Bivariant	0.694	0.567	0.675
Lumbar lordosis	Spearman’s Rho	0.036	− 0.211	0.218
	Bivariant	0.899	0.451	0.435
Sacral slope	Spearman’s Rho	*− 0.532*	− 0.395	0.175
	Bivariant	**0.041**	0.145	0.532
Pelvic tilt	Spearman’s Rho	0.433	0.455	− 0.44
	Bivariant	0.122	0.102	0.115
Pelvic incidence	Spearman’s Rho	0.332	0.389	− 0.458
	Bivariant	0.246	0.169	0.1

^*^The correlation is at *p *value of < 0.05 significant (bivariant); *p *values (bold) with corresponding correlation coefficients (italic)

## Discussion

Our data provide improved outcome after reduction surgery in low-grade spondylolisthesis in a cohort of 32 patients. All clinical outcome parameters improved significantly to preoperative values. By correlation of sagittal balance parameters to clinical outcome scores, we found correlation between sacral inclination, sacral slope and sagittal rotation to postoperative COMI and ODI scores at 3-year follow-up. Change was found in outcome of the 3-year follow-up data compared to our previously published 2-year follow-up [20].

Lately, Le Huec et al. [21] published the benefit of restoration of lumbar lordosis leading to rotation of the pelvis, thus, resulting in restoration of spinopelvic parameters. With the balanced pelvis, due to reduction and fusion surgery, clinical outcome of patients was improving. To the contrary, recent findings suggest no advantage of reduction when the pelvis is already balanced [22]. Excluding the pelvic factors, several publications postulate a benefit of reduction of anterior displacement. Given these data describing benefits of restoration of lumbar lordosis, the amount of restoration of anterior displacement and the benefit in extent of preoperative slippage in degenerative spondylolisthesis is still divisive. In their prospective study, Wegmann et al. [17] showed a mild correlation between reduction of slippage and better clinical outcome in 40 patients with posterior interbody lumbar fusion.

In concordance with these findings, Kawakami et al. [23] reported a significantly better outcome in patients with restoration of lumbar lordosis suffering from major preoperative slippage in degenerative spondylolisthesis. Contrary, several studies and systematic reviews reject any improvement in clinical outcome by restoring sagittal parameters by far [24, 25]. In their systematic review, Rhee et al. [26] analyzed 13 studies in degenerative spondylolisthesis and described no benefit of reduction in monosegmental fusion surgery. Moreover, Lian et al. [16] published cases with mild degenerative spondylolisthesis with minor anterior displacement had no benefit of reduction. This is contradicting our recent findings, given a correlation between restoration of lumbar lordosis (sacral slope; sacral inclination) and improvement in COMI score.

When we first published the data of the 2-year follow-up, a significant benefit in mild spondylolisthesis (Meyerding grades I and II) in clinical outcome (COMI, ODI, VAS) after surgery was detected. However, no significant correlation was observed in improvement of clinical outcome and restoration of radiographic parameters. A significant correlation between the increase of sagittal rotation and improvement of the EQ-5D could only be shown 1 year after surgery, anticipating correction of sagittal rotation could improve the alignment of spine and pelvis leading to better clinical outcome. However, we could not further demonstrate this correlation in our 3-year follow-up. On the contrary, now we could demonstrate strong correlation between improvement in COMI score and both sacral inclination (r*0.586) and sacral slope (r*0.532). Sacral inclination is a key parameter in sagittal balance and contributes in the development of spondylolisthesis. However, sacral inclination can be restored to physiological levels influencing the pelvic incidence and therefore a balance in sagittal spinopelvic alignment can achieved. Furthermore, spinopelvic weight balance which is addressed by surgical reduction of anterior displacement may lead to less lordosis in lumbar spine and an improvement of spinopelvic harmony yielding lower levels of pain levels, thus showing better clinical outcome. Sacral slope only showed minimal change in the follow-up, but a strong correlation was found with improvement in COMI score suggesting better spinopelvic alignment after surgical reduction. However, the correlation of sagittal spinal parameters and improvement in outcome may be due to better spinopelvic balance, already shown by previous findings [21]. Moreover, a correlation between ODI and reduction of sagittal rotation was found in the 3 years’ follow-up (r*0.517; *p* < 0.05). With less sagittal rotation, the weight distribution of the lumbar spine could be better balanced resulting in less force on each vertebrae and vertebral discs. However, in low-grade spondylolisthesis, the impact of sagittal rotation and anterior displacement to spinopelvic factors is far less immanent than compared to higher grades of spondylolisthesis. Regarding surgical procedure, due to a greater (bilateral) removal of dorsal vertebral structures, PLIF might allow further modulation of sacral inclination and lumbar lordosis [27]. However, the influence of the procedure might not be significant. In our cohort, the minimal number in TLIF procedures did not allow for further subanalysis. Moreover, the number of fused segments might influence restoration of sagittal parameters significantly due to higher potential of correction by either compression or distraction [28].

With this finding, we hypothesize an effect in postoperative mid-term progress by stabilization of spinopelvic alignment, higher muscular strength by rehabilitation and adoption of sagittal balance of the patients over time. Regarding literature review and our data, sagittal balance may only be partly crucial for clinical outcome (ODI, COMI, EQ-5D). It may rather be postulated, that prevention of adjacent segment disease (ASD) in the postoperative process is key to maintain good clinical outcome. Therefore, restoration of sagittal balance may support better biomechanical stability throughout balanced weight distribution and function of spinal and pelvic interaction resulting in lower rate of ASD.

This study has several limitations as it is a monocentric prospective study and performance bias of surgeons might have influenced the results as well as drop-outs within time led to a small total of patients. Due to reduction of radiation, only lumbar radiographs were performed instead of full spine radiographs; thus, classification of Roussouly could not be included. Furthermore, no differentiation was made regarding segmental fusion of L4/5 or L5/S1. Nevertheless, our study provides good clinical data in a 3-year follow-up in low-grade spondylolisthesis and raises controversies as mid-term effects in reduction of anterior displacement and restoration of sagittal balance might be seen with improvement of clinical outcome.

## Conclusions

In our findings, reduction of sagittal rotation and sacral inclination showed significant correlation showing improvement of ODI and COMI scores in a 3-year follow-up. However, controversial results regarding the impact of reduction and restoration of sagittal parameters in regards of clinical outcome are published constantly. Certain additional factors such as the grade of slippage in spondylolisthesis, pelvic factors and surgical factors might contribute in a significant manner.
